# Clitoral sizes and anogenital distances in term newborns in Nigeria

**DOI:** 10.1186/s13633-019-0069-6

**Published:** 2019-12-05

**Authors:** Adesola Olubunmi Adekoya, Musili Bolanle Fetuga, Olumide Olatokunbo Jarrett, Tinuade Adetutu Ogunlesi, Jean-Pierre Chanoine, Abiola Omobonike Adekoya

**Affiliations:** 1 Department of Paediatrics, Babcock University Teaching Hospital and Ben Carson School of Medicine, Babcock University, Ilishan-Remo, Ogun State Nigeria; 2 Department of Paediatrics, Olabisi Onabanjo University Teaching Hospital, Sagamu, Ogun State Nigeria; 3 Department of Paediatrics, University College Hospital and College of Medicine, University of Ibadan, Ibadan, Oyo State Nigeria; 4 Endocrinology and Diabetes Unit, British Columbia’s Children’s Hospital, University of British Columbia, Vancouver, BC Canada; 5 Department of Radiology, Olabisi Onabanjo University Teaching Hospital, Sagamu, Ogun State Nigeria

**Keywords:** Anus-clitoris distance, Anus-fourchette distance, Clitoral length, Clitoromegaly, Nigeria

## Abstract

**Background:**

Previous studies suggest significant ethnic and racial differences in clitoral sizes and anogenital distances in the newborn. This study aimed to document normative data on clitoral sizes and anogenital distances of apparently normal term female infants in Sagamu.

**Methods:**

The study was a multi-center, cross-sectional descriptive research carried out among 317 female term infants within the first 72 h of life. Interviewer-based questionnaire was applied to obtain sociodemographic data, pregnancy and birth history. A sliding digital caliper was used for measurement. Data analysis was with SPSS version 20.0.

**Results:**

The mean clitoral length was 6.7 ± 1.6 mm while the mean clitoral width was 5.6 ± 0.8 mm. The mean fourchette-clitoris distance, anus-clitoris distance and anus-fourchette distance were 21.9 ± 2.1 mm, 35.5 ± 2.5 mm and 17.0 ± 2.6 mm respectively. The anus-clitoris and anus-fourchette distances significantly correlated with the anthropometric parameters while the clitoral measurements did not.

**Conclusion:**

The mean values recorded in this study were higher than observed in most previous studies. This simple, affordable and non-invasive evaluation could aid early diagnosis and treatment of female infants with potentially harmful conditions such as congenital adrenal hyperplasia.

## Background

A common practice globally after the delivery of a baby is to identify the sex of the baby even if there is a foreknowledge of this via a prenatal obstetric ultrasound scan. A delay, for any reason in assigning the sex could be distressing for the doctor, the midwife as well as having a significant psychosocial effect on the parents and family [1]. Apart from sex assignment, it is essential that genitalia be routinely and carefully examined with other anthropometric parameters in the newborn as this may aid early detection of life-threatening conditions such as congenital adrenal hyperplasia (CAH) [2].

In-utero exposure of the female fetus to excess androgenic substances may result in varying levels of genital ambiguity and increased ano-genital distances (AGD) [3, 4]. Clitoromegaly in a newborn female could be due to foetal or maternal causes and should be regarded as potentially pathologic [5]. These observations indicate the importance of clitoral and ano-genital measurements as markers of in-utero exposure to androgens.

Anthropometric measurements in the paediatric age group are most useful when there are acceptable reference values to compare them with. This is best applied in a local context as ethnic and racial differences in clitoral sizes and AGD have been reported in previous studies [6–10]. In countries where routine newborn screening for congenital adrenal hyperplasia is not practiced, the availability of references for clitoral sizes and ano-genital distances may be helpful in assessing virilised genetic females and investigating them further. In a recent Nigerian publication, four females out of 24 children (16.7%) with different types of CAH presented in the neonatal period with ambiguous external genitalia in a 10-year review of CAH [11].

Globally, only few authors measured both clitoral sizes and AGD at the same time in the newborn. In Nigeria, there are isolated studies that measured either clitoral sizes or AGD in newborn females [6–8]. The aim of this study was therefore to determine normative data of clitoral sizes and AGD among term female newborn infants in a Nigerian population.

## Methods

This cross-sectional and multi-centered study was conducted in Sagamu town in Southwest Nigeria. The subjects were recruited from the state-owned university teaching hospital and four other health facilities including two primary health centers and two private hospitals, which were randomly selected.

The minimum sample size was calculated using the formula [12] *N* = (Zσ /E)^2^ where Z is the value from the standard normal distribution reflecting the confidence level (Z = 1.96 for 95%), σ was the standard deviation obtained in reference study and E, the desired margin of error. Mean AGD from reference study [7] was AGD of 13.89 ± 2.6 mm. A margin of error of 1/3 mm (0.33 mm) was desired {*N* = (1.96 × 2.6/0.33) [2] = 239}.

Term, healthy babies aged 0–72 h and delivered by spontaneous vertex delivery were included in the study. The exclusion criteria included the presence of dysmorphic features, ambiguity of the genitalia, known maternal ingestion of substances that are deemed by the investigator to have androgenic effects, features of masculinization in pregnancy and if either of the parents was not from any tribe in Nigeria. Information about the family and the pregnancy were obtained from the mothers using interviewer-based questionnaire. The maternal last regular menstrual period or an ultrasound scan done before 20 weeks was used to estimate the gestational age of the babies. The New Ballard score [13] was used if the difference between the two was greater than 2 weeks.

### Measurements

All clitoral and anogenital measurements were taken by one examiner (A.O.A.). The same equipment was used for all babies to prevent errors due to inter-equipment variation. An electronic weighing scale (Seca® 384; Hamburg, Germany) and a portable infantometer (Seca® 416; Hamburg, Germany) were used to measure the weight and length respectively. The head and chest circumferences were measured using a non-stretchable, plastic tape according to standardized protocols.

Each baby was exposed and placed in the dorsal decubitus position with the assistant gently flexing the knees while applying light pressure on the hips. The distances from the centre of the anus to the beginning of the mucosa of the posterior fourchette (anus-fourchette distance, AFD), from the centre of the anus to the base of the clitoris (anus-clitoris distance, ACD) and from the beginning of the mucosa of the posterior fourchette to the base of the clitoris (fourchette-clitoris distance, FCD) were measured [14].

The clitoral length was measured from the pubic ramus to the tip of the glans clitoris after parting the labia majora and gently retracting the hood of the clitoris [4] The clitoral width was measured by taking the transverse diameter at the middle of the clitoris using the same caliper [4].

All clitoral and anogenital measurements were taken twice using an adjustable caliper [VWR digital 152 cm (6 in.) caliper -stainless steel- model 62,379–531] which is calibrated to the nearest millimeter. Throughout the examination and measurements of all the infants in this study, the mother, or the father, or a close relative was present to observe every action taken on their baby. The average of the two measurements was adopted. The intraclass correlation coefficient for FCD, ACD, AFD, CL and CW were 0.901, 0.971, 0.976, 0.967 and 0.936 respectively.

Data was analysed using the Statistical Package for the Social Sciences (SPSS) for Windows version 20.0 IBM Corporation, Chicago, IL, USA. Where applicable, means and standard deviations, proportions and percentages were calculated. Pearson correlation coefficient (r) was used to determine the relationship between continuous variables. Statistical significance was set at *p* values less than 0.05 in a two-tailed test.

## Results

A total of 317 term female newborns were included in the study which spanned between April 2014 and March 2015. The mean age at measurement was 21.3 ± 9.0 h (4–66). The mean (±SD) gestational age, weight, length, head and chest circumference were 39.3 ± 1.1 weeks, 3.0 ± 0.3 kg, 48.6 ± 1.9 cm, 34.5 ± 1.2 cm and 32.3 ± 1.7 cm respectively. The distribution, range, mean values and the derived percentiles of the clitoral and ano-genital measurements are presented in Table 1. The mean (±SD) values of AF/AC, FC/AC AF/FC ratios were 0.48 ± 0.05, 0.62 ± 0.06 and 0.78 ± 0.14 respectively.

**Table 1:** Range, mean values and percentiles of clitoral and ano-genital measurements

	Range	Mean + SD	Percentiles		
			3rd	50th	97th
CL (mm)	3.1–12.5	6.7 ± 1.6	3.8	6.5	9.6
CW (mm)	3.6–8.4	5.6 ± 0.8	4.2	5.6	7.3
ACD (mm)	29.5–41.9	35.5 ± 2.5	31.2	35.7	39.7
AFD (mm)	10.0–24.8	17.0 ± 2.6	12.7	16.7	21.3
FCD (mm)	15.9–39.1	21.9 ± 2.1	18.6	21.8	25.7

*CL* Clitoral length, *CW* Clitoral width, *ACD* Anus-clitoris distance, *AFD* Anus-fourchette distance, *FCD* Fourchette-clitoris distance

Only the ACD and AFD significantly correlated with the anthropometric variables. There was a significant but negative correlation between AFD and FCD while ACD positively correlated with both AFD and FCD. Clitoral length correlated positively with clitoral width. Other details are as shown in Table 2.

**Table 2:** Pearson’s Correlation of clitoral and ano-genital measurements with anthropometry

		CL	CW	ACD	AFD	FCD
BL	r	0.019	−0.098	0.228	0.170	0.078
	*p*	0.734	0.081	**0.000**	**0.002**	0.165
BW	r	−0.101	− 0.098	0.415	0.325	0.095
	*p*	0.073	0.081	**0.000**	**0.000**	0.090
HC	r	−0.043	−0.095	0.269	0.223	0.094
	*p*	0.447	0.092	**0.000**	**0.000**	0.096
CC	r	−0.079	−0.092	0.298	0.229	0.067
	*p*	0.161	0.101	**0.000**	**0.000**	0.231
CL	r		0.386	− 0.135	− 0.112	0.070
	*p*		**0.000**	**0.016**	**0.046**	0.213
CW	r			0.019	− 0.086	0.095
	*p*			0.740	0.128	0.092
ACD	r				0.533	0.403
	*p*				**0.000**	**0.000**
AFD	r					− 0.196
	*p*					**0.000**

*BL* Birth length, *BW* Birth weight, *HC* Head circumference, *CC* Chest circumference, *CL* Clitoral length, *CW* Clitoral width, *ACD* Anus-clitoris distance, *AFD* Anus-fourchette distance, *FCD* Fourchette-clitoris distance, *r* Correlation coefficient. *p <* *0.05 is significant*

## Discussion

We established normative data which will aid the identification and further evaluation of newborn females with abnormal clitoral sizes and ano-genital distances for the possible aetiology, as well as management in Southwest of Nigeria.

An abnormally-sized clitoris may either be enlarged or small and defined as clitoromegaly or microclitoris respectively, with the former discussed far more than the latter in the literature. Using the definition of mean + 2 SD, we define clitoromegaly as a clitoris greater than 9.9 mm in length. In many neonates, no cause is found (idiopathic clitoromegaly) [15] but, it can also reflect exposure of the fetus to fetal or maternal androgens. Congenital adrenal hyperplasia is the commonest cause of pathological clitoromegaly in the newborn [6]. Other causes of clitoromegaly include conditions such as fetal aromatase deficiency [16] maternal and placental factors such as luteoma of pregnancy, ovarian tumors and placental aromatase deficiency [17].

In the absence of systematic neonatal CAH screening, identification of clitoromegaly (of genital ambiguity) may be the earliest sign of CAH at birth. This is a reminder of the importance of careful examination of the genitalia in the neonate, which is not routinely performed in many parts of Africa. It provides a unique opportunity to identify clitoromegaly and to perform an early diagnosis of CAH in affected infants [2]. Clitoromegaly may also be part of complex disorders, including Russell-Silver Syndrome, Beckwith-Wiedemann Syndrome and Fraser syndrome [15].

On the other hand, microclitoris is more difficult to recognize because the low end of the normal spectrum (3rd percentile) is already very small (3.6 mm). Using the definition of mean – 2SD, we define microclitoris as a clitoris less than 3.2 mm in length. Aetiology of microclitoris in the newborn include Prader-Willi syndrome [18].

The conventional ano-genital ratio, which corresponds to the AF/AC ratio is considered as a marker for the degree of virilization. A value greater than 0.5 suggests posterior labial fusion and virilization [16]. Consistent with this data, the ratio observed in this study was 0.48, slightly higher than the ratio observed in American (0.37 ± 0.07) [14] and Indian infants (0.34 ± 0.07) [19].

The effects of environmental endocrine disrupting chemicals on the developing genitalia in newborn females have been observed. Shorter ano-genital distances (AGD) were documented in infants of mothers with high bisphenol A in early trimester [20] while longer AGD was reported in those with maternal polycystic ovarian syndrome (PCOS) [21]. Therefore, the evaluation for abnormal clitoral sizes and AGD in the newborn requires detailed maternal history.

There was no significant correlation between clitoral length, width and the anthropometric measurements in the present study. These findings are in agreement with some [4] but not all reports [3]. Only the ACD and AFD were positively correlated with anthropometric parameters, contrasting with findings by Ozkan et al [22] who found that all three ano-genital measurements increased as the weight, length and head circumference increased.

The mean clitoral length in the present study is higher than documented in some countries [3, 4, 8, 10]. Similarly, the AFD was higher than previously reported [8, 10, 23, 24]. The same observation was reported in male newborns in the same location [25]. Although these various observations suggest that newborn clitoral length and anogenital distances may differ according to ethnicity, they may also reflect differences in the methodology.

A strength of our study is that we ensured that all participants belonged to a Nigerian tribe, meaning that our results only reflect the characteristics of this part of Africa. Our study also has limitations. First, all measurements were performed by one investigator. Therefore, we cannot provide any information on interobserver variability which may affect the interpretation of measurements performed by other clinicians. Second, the investigator only assessed whether mothers were taking androgenic substances through history and could not verify through chart review. It is possible that the mother did not report all substances consumed.

## Conclusions

Our study provides reference values on clitoral sizes and ano-genital distances in Nigerian female newborns. Clinically, the most important measurement is the length of the clitoris as clitoromegaly may reflect excessive androgen exposure. Our study also serves as a reminder that neonatal genital examination, which is not routinely performed in many settings in Africa, should be part of a systematic, routine neonatal examination by birth attendants. This simple, affordable and non-invasive evaluation could aid early diagnosis and treatment of female infants with potentially harmful conditions such as congenital adrenal hyperplasia, particularly when newborn screening is not available.

## Supplementary information

**Additional file 1.** Clitoral and anogenital measurements.

## Data Availability

All data analysed (Clitoral and anogenital measurements) during this study are included in this article as Additional file 1.

## Abbreviations

ACD: Anus-clitoris distance
AFD: Anus-fourchette distance
CL: Clitoral length
CW: Clitoral width
FCD: Fourchette-clitoris distance
OOUTH: Olabisi Onabanjo University Teaching Hospital
